# Clinical and laboratory features of hypercoagulability in COVID-19 and other respiratory viral infections amongst predominantly younger adults with few comorbidities

**DOI:** 10.1038/s41598-021-81166-y

**Published:** 2021-01-19

**Authors:** Chuen Wen Tan, Jing Yuan Tan, Wan Hui Wong, May Anne Cheong, Ian Matthias Ng, Edwin Philip Conceicao, Jenny Guek Hong Low, Heng Joo Ng, Lai Heng Lee

**Affiliations:** 1 Department of Haematology, Singapore General Hospital, 20 College Road, Singapore, 169856 Singapore; 2 SingHealth Internal Medicine Residency, Singapore General Hospital, Singapore, Singapore; 3 Department of Infection Prevention and Epidemiology, Singapore General Hospital, Singapore, Singapore; 4 Department of Infectious Diseases, Singapore General Hospital, Singapore, Singapore; 5 Programme in Emerging Infectious Diseases, Duke NUS Medical School, Singapore, Singapore

**Keywords:** Infectious diseases, Viral infection, Biomarkers, Epidemiology

## Abstract

COVID-19 caused by Severe Acute Respiratory Syndrome Coronavirus-2 (SARS-CoV-2) and other respiratory viral (non-CoV-2-RV) infections are associated with thrombotic complications. The differences in prothrombotic potential between SARS-CoV-2 and non-CoV-2-RV have not been well characterised. We compared the thrombotic rates between these two groups of patients directly and further delved into their coagulation profiles. In this single-center, retrospective cohort study, all consecutive COVID-19 and non-CoV-2-RV patients admitted between January 15th and April 10th 2020 were included. Coagulation parameters studied were prothrombin time and activated partial thromboplastin time and its associated clot waveform analysis (CWA) parameter, min1, min2 and max2. In the COVID-19 (n = 181) group there were two (1.0 event/1000-hospital-days) myocardial infarction events while one (1.8 event/1000-hospital-day) was reported in the non-CoV-2-RV (n = 165) group. These events occurred in patients who were severely ill. There were no venous thrombotic events. Coagulation parameters did not differ throughout the course of mild COVID-19. However, CWA parameters were significantly higher in severe COVID-19 compared with mild disease, suggesting hypercoagulability (min1: 6.48%/s vs 5.05%/s, *P* < 0.001; min2: 0.92%/s^2^ vs 0.74%/s^2^, *P* = 0.033). In conclusion, the thrombotic rates were low and did not differ between COVID-19 and non-CoV-2-RV patients. The hypercoagulability in COVID-19 is a highly dynamic process with the highest risk occurring when patients were most severely ill. Such changes in haemostasis could be detected by CWA. In our population, a more individualized thromboprophylaxis approach, considering clinical and laboratory factors, is preferred over universal pharmacological thromboprophylaxis for all hospitalized COVID-19 patients and such personalized approach warrants further research.

## Introduction

Towards the end of April 2020, investigators were reporting the association of Coronavirus Disease-19 (COVID-19) with increased incidence of thrombotic events. Initially confined to intensive care units (ICU) and critically ill COVID-19 patients, similar findings were subsequently also noticed in non-critically ill patients^1,2^. Emerging autopsy-derived histopathological evidence have confirmed the presence of pulmonary vasculature microthrombosis in deceased patients^3,4^. Thrombotic complication is however not unique to COVID-19. Other non-CoV-2 respiratory viral (non-CoV-2-RV) infections have also been associated with both arterial^5,6^ and venous^7^ thrombosis.

The thrombotic manifestations in COVID-19 patients have led to both the American Society of Hematology (ASH)^8^ and International Society on Thrombosis and Hemostasis (ISTH)^9^ recommending that all hospitalized patients including the non-critically ill, receive venous thromboembolism (VTE) thromboprophylaxis with low molecular weight heparin (LMWH) or fondaparinux. It is uncertain if such recommendations should be universally adopted as there is significant heterogeneity in reported thrombotic rates as most studies were carried out on critically ill COVID-19 patients^10^. Additionally, baseline VTE risk is known to differ between geographical regions, race and ethnicity^11,12^. As the absolute risk of bleeding increases by around 0.5% with thromboprophylaxis^13^, stratifying COVID-19 patients with high risk of VTE for thromboprophylaxis is crucial to optimize the risk benefit ratio. Measurable laboratory parameters such as elevated D-dimer and fibrinogen levels have been reported to be associated with thrombosis in COVID-19. Less accessible global hemostatic assays such as thromboelastogram and clot waveform analysis (CWA) have also demonstrated hypercoagulability, albeit in small groups of critically ill COVID-19 patients^14,15^.

In this study, we aimed to determine the thrombotic rates in consecutive COVID-19 patients compared to patients with non-CoV-2-RV infections and to evaluate the dynamic haemostatic changes in both groups of patients using global coagulation assays.

## Methods

This single-center study was conducted at Singapore General Hospital, a 1700-bedded tertiary academic center.

For our first objective, we performed a retrospective cohort study to determine thrombotic event rates in all consecutive COVID-19 patients compared to patients diagnosed with non-CoV-2-RV from January 17th to April 10th 2020. During this period, all patients who fulfilled our hospital’s COVID-19 suspect criteria were admitted regardless of disease severity and tested for both COVID-19 and other respiratory viral pathogens^16^. COVID-19 testing was performed with reverse transcriptase polymerase chain reaction (rt-PCR) on RNA extracted from either oropharyngeal and/or nasopharyngeal swabs specimens. Testing for other respiratory viral pathogens was performed using a 16-target respiratory viral PCR assay (Respiratory syncytial virus A/B, Influenza A/B, Parainfluenza viruses 1–4, Metapneumovirus, Rhinovirus A/B/C, Human Coronavirus OC43/229E/NL63, Adenovirus, Human Enterovirus, Human Bocavirus 1–4) on oropharyngeal specimens. Exemption of infroemd consent was granted by Singhealth Centralised Institutional Review Board as this study only involved collection and analysis of routine clinical data (CIRB no. 2020/2535). Study data was obtained from our Hospital’s Department of Infection Prevention and Epidemiology database. This database consists of de-identified data collected from all confirmed and suspect COVD-19 cases admitted to our hospital during this pandemic period. Data on demographics, co-morbidities, laboratory coagulation profile parameters, and anti-platelet/anticoagulation medications were obtained. Information on modality of thromboprophylaxis (mechanical vs routine chemical) initiated, thrombotic events during hospitalization, the need for high dependency unit (HDU) and intensive care unit care (ICU), length of stay in ICU and length of hospitalization was also obtained. Need for oxygen supplementation during hospital admission was also collected and was used as a surrogate measure for disease severity in our analysis. We included both arterial and venous thrombotic events. Venous thrombotic events were defined as any venous thromboembolism including deep vein thrombosis, pulmonary embolism and thrombosis of other sites, which were objectively confirmed on radiological imaging after initial clinical suspicion by attending physicians. Arterial events were defined as either myocardial infarction or stroke.

For our second objective, laboratory coagulation tests data were pooled from two sources: a) both COVID-19 and non-CoV-2-RV patients as studied above and b) COVID-19 patients from our hospital’s infectious disease department novel pathogen study database (CIRB no. 2018/3045) censored at 1st June 2020. This database is part of a prospective study to characterize emerging infectious diseases and was approved by Singhealth Centralised Institutional Review Board (CIRB no. 2018/3045). We collected the following coagulation data: prothrombin time (PT), activated partial thromboplastin time (aPTT) and its associated clot waveform analysis (CWA), fibrinogen and D-dimer. Patient samples and data were anoynymised by research staff and clinicians involved in the novel pathogen study database project. We complied with all relevant ethical regulations.

Coagulation tests taken when patients were on anticoagulants or on citrate-based haemodialysis and patients with active malignancy or pregnancy were excluded from any inter-individual comparison and analysis. APTT-based CWA were generated during the analysis of the standard aPTT assay triggered with Dade Actin FSL reagent (Siemens Healthcare, Marburg, Germany) and retrieved from CS2100i automated coagulation analysers (Sysmex Corporation, Kobe, Japan). The three CWA parameters of interest were min1, min2 and max2 denoting the maximum velocity, maximum acceleration and maximum deceleration of the clot formation process, respectively.

### Statistical analysis

Categorical variables were presented as frequencies (percentages) and continuous variable was presented as median (interquartile range) due to its skewed distribution. Univariate analysis was performed using chi square test for categorical variables and Mann Whitney U test for continuous variables. Correlation analysis was performed using Spearman’s rank correlation coefficient (R_S_). All tests were two sided, with p-values of *P* < 0.05 considered statistically significant. Data analysis was performed using SPSS 25.0 (IBM SPSS statistics, IBM Corporation, USA) software.

## Results

During the study period, a total of 181 patients testing positive for SARS-CoV-2 and 165 patients positive for non-CoV-2-RV via rt-PCR testing. The respiratory viruses were rhinovirus (n = 65), influenza A and B (n = 46), adenovirus (n = 13), human coronavirus 229E/NL63/OC43 (n = 15), human enterovirus (n = 3), metapneumovirus (n = 6), parainfluenza virus 1 to 4 (n = 11) and respiratory syncytial virus (n = 6). Among the 186 COVID-19 patients, four patients were co-infected with other respiratory viruses and were excluded from analysis. Age and comorbidities were not significantly different between the two groups (Table 1). Compared to the non-CoV-2-RV arm, there were significantly higher proportion of male patients and patients of Indian ethnicity in the COVID-19 arm. Majority (94%) of our COVID-19 patients were managed in the general isolation wards and had relatively mild infection as reflected by low proportions requiring any form of oxygen supplementation (11%). COVID-19 patients had longer length of hospitalization and more required ICU support. Mortality rate was low in both groups. The two deaths in our COVID-19 cohort were due to severe COVID-19 pneumonia with multi-organ failure.

**Table 1:** Clinical characteristics, thrombotic events and outcomes of patients with SARS-CoV-2 and non-CoV-2 respiratory viruses.

	SARS-CoV-2 (n = 182)	Non-CoV-2 respiratory viruses (n = 165)	*P* values
**Demographics**			
Age, Median (IQR)	37 (30.51)	35 (29.51)	0.122
Gender—no.(%)			< 0.001
Male	133 (73.1)	87 (52.7)	
Female	49 (26.9)	78 (47.3)	
Race—no.(%)			< 0.001
Chinese	63 (34.6)	74 (44.8)	
Malay	6 (3.3)	31 (18.8)	
Indian	81 (44.5)	35 (21.2)	
Others	32 (17.6)	25 (15.2)	
**Comorbidities**—no.(%)			
Hypertension	44 (24.2)	28 (17.0)	0.098
Hyperlipidemia	15 (8.20)	24 (18.50)	0.063
Diabetes Mellitus	10 (5.5)	22 (13.3)	0.012
Ischemic Heart Disease	6 (3.30)	13 (7.90)	0.061
Prior stroke	4 (2.20)	5 (3.0)	0.626
Renal disease	1 (0.50)	10 (6.10)	0.003
Chronic lung disease	18 (9.90)	15 (9.10)	0.800
Liver disease	0 (0.00)	3 (1.80)	0.106
Active malignancy	0 (0.00)	3 (1.80)	0.106
Charlson Comorbidity index			
Median (IQR)	0 (0.1)	0 (0.1)	0.39
0–2 points	167 (91.8)	145 (87.9)	0.231
≥ 3 points	15 (8.2)	20 (12.1)	
**Existing antithrombotic agents**—**no.(%)**			
Antiplatelet	8 (4.4)	7 (4.2)	1.0
Anticoagulation	1 (0.5)	1 (0.6)	1.0
**Thrombotic outcomes**			
Arterial thrombosis—no.(%)	2 (1.1)	1 (0.6)	1.0
Venous thrombosis—no.(%)	0	0	–
Event/1000 patient days	1.0	1.8	0.6308
**Outcomes**			
Required O2 supplementation—no.(%)	20 (11.0)	8 (4.8)	0.035
HD—no.(%)	2 (1.1)	0 (0)	0.500
ICU—no.(%)	9 (4.9)	2 (1.2)	0.047
LOS in ICU, median (IQR)	20 (9.27)	5 (3.7)	0.073
Total patient days in ICU	160	10	–
LOS, Median (IQR)	7.5 (6.13)	3 (2.3)	< 0.001
Total patient days in hospital	1979	554	–
Death—no.(%)	2 (1.1)	1 (0.6)	1.0

### Thrombotic outcomes

In both groups, all general ward patients were not given routine thromboprophylaxis. There was no standardized hospital practice with regards to pneumatic calf pumps for general ward patients and thus such data was unavailable. Patients in HDU were only on mechanical thromboprophylaixs (TED stockings). In contrast, patients in ICU were all started on prophylactic subcutaneous enoxaparin 40 mg once a day (or 20 mg once a day for patients with renal failure) unless contraindicated, together with pneumatic calf pumps. Amongst our COVID-19 ICU patients, two (22.2%) out of nine patients were not on prophylactic thromboprophylaxis because of thrombocytopenia and anemia respectively. There were two arterial thrombotic events in the COVID-19 group, both of which occurred while patients were in ICU. This corresponded to a 22.2% incidence amongst COVID-19 ICU patients and 1.1% incidence amongst all COVID-19 patients. On the other hand, there was only one arterial thrombotic event (0.6% incidence) in the non-CoV-2-RV group and this occurred in a severely ill patient. The thrombotic rates in COVID-19 and non-CoV-2-RV patients adjusted for the duration of hospitalization translated to 1 per 1000 patient-days and 1.8 per 1000 patient-days respectively. There were no VTE events in both groups. Only one patient had disseminated intravascular coagulopathy (DIC) by ISTH criteria^17^ and sepsis induced coagulopathy (SIC) by criteria proposed by Iba et al.^18^. This occurred on day 26 of symptoms and day 21 of ICU stay at which time there was an ongoing component of ventilator associated pneumonia and there was no occurrence of thrombotic event during the study period.

Both cases of arterial thrombosis in the COVID-19 group were non-ST elevation myocardial infarction (NSTEMI). Both occurred early in the course of disease, at day 9 and day 12 of symptom onset respectively. The arterial thrombosis in our non-CoV-2-RV cohort was also a NSTEMI, developing on day 4 of admission. All cases were reviewed by our hospital’s cardiologist and were started on antiplatelet therapy. No diagnostic coronary angiogram was done for the cases at time of diagnosis. All of them were on prophylactic enoxaparin at the time of thrombosis. There was only one major bleeding event due to intracranial haemorrhage which occurred in the ICU in our entire cohort.

### Hemostatic assays

The coagulation profiles and CWA parameters were analysed. 42 COVID-19 and 5 non-CoV-2-RV patients had only a single data point. 14 COVID-19 patients had data available on two or more time points while only two of the non-CoV2-RV patients had serial coagulation profile data, both of whom were critically ill. No biphasic waveform was noted in all the clot waveform curves analysed. The key findings are summarized here:

- Coagulation functions remained fairly stable in mild COVID-19 and not significantly different from baseline (convalescent state) within the same individuals (Table 2)

**Table 2:** Paired comparison analysis of coagulation data (PT, aPTT and aPTT-based clot waveform analysis profiles) of patients with mild COVID-19^. These are intra-individual comparison of their serial coagulation data.

Coagulation tests	1st time point (n = 10)		2nd time point (n = 10)		*P* value
	Median (IQR)	Range	Median (IQR)	Range	
APTT, s	31.98 (3.65)	28.75–36.05	30.70 (3.70)	25.80–36.90	0.066
Min1, %/s	5.04 (1.76)	3.67–7.04	5.16 (3.53)	3.15–8.67	0.386
Min2, %/s^2^	0.71 (0.24)	0.57–1.05	0.77 (0.62)	0.47–1.35	0.286
Max2, %/s^2^	0.56 (0.18)	0.44–0.85	0.59 (0.53)	0.35–1.11	0.259
PT, s	10.20 (0.70)	9.70–10.90	10.30 (0.80)	9.50–11.40	0.777
Days since symptoms onset	7 (5)	5–17	10.5 (8)	7–19	0.005

	1st time point (n = 7)		3nd time point (n = 7)		
APTT, s	32.25 (2.35)	28.75–34.85	30.40 (3.25)	28.00–32.45	0.051
Min1, %/s	4.80 (0.76)	4.13–7.04	4.25 (1.04)	2.85–8.17	0.236
Min2, %/s^2^	0.70 (0.14)	0.61–1.05	0.58 (0.21)	0.45–1.29	0.310
Max2, %/s^2^	0.54 (0.12)	0.47–0.80	0.44 (0.24)	0.35–1.05	0.351
PT, s	10.30 (0.40)	9.70–10.80	10.80 (0.80)	10.00–11.40	0.058
Days since symptoms onset	7 (9)	6–17	14 (8)	11 –22	0.018

	1st time point (n = 4)		Convalescence (n = 4)		
APTT, s	31.05 (2.99)	30.05–34.00	27.80 (2.40)	27.40–30.50	0.068
Min1, %/s	5.79 (1.76)	5.07–7.04	5.90 (2.62)	4.76–7.53	0.715
Min2, %/s^2^	0.90 (0.261)	0.78–1.05	0.93 (0.43)	0.76–1.22	0.465
Max2, %/s^2^	0.71 (0.259)	0.57–0.85	0.76 (0.39)	0.61–1.04	0.068
PT, s	10.40 (0.70)	9.80–10.70	10.25 (1.20)	9.70–11.30	1.000
Days since symptoms onset	8.5 (9)	4–15	48 (67)	22–99	0.068

* No D-dimer and fibrinogen data presented as not more than 2 pairs of these results were available.

^ Mild COVID-19 defined as patients who did not require supplementary oxygen support throughout the course of infection.

In COVID-19 patients who remained clinically well without a need for supplemental oxygen, intra-individual paired comparison of PT, aPTT and CWA data across various time points were not significantly different throughout the course of the disease. Paired comparison between the initial and convalescent coagulation tests also did not show remarkable alteration.

In addition, inter-individual comparison between the presenting coagulation assays taken from patients with mild COVID-19 and coagulation assays taken at convalescent phase also supported this observation – although aPTT was shorter in convalescent phase, CWA profiles and PT were not significantly different between the two groups (Supplementary Table 1).

**Figure 1:**
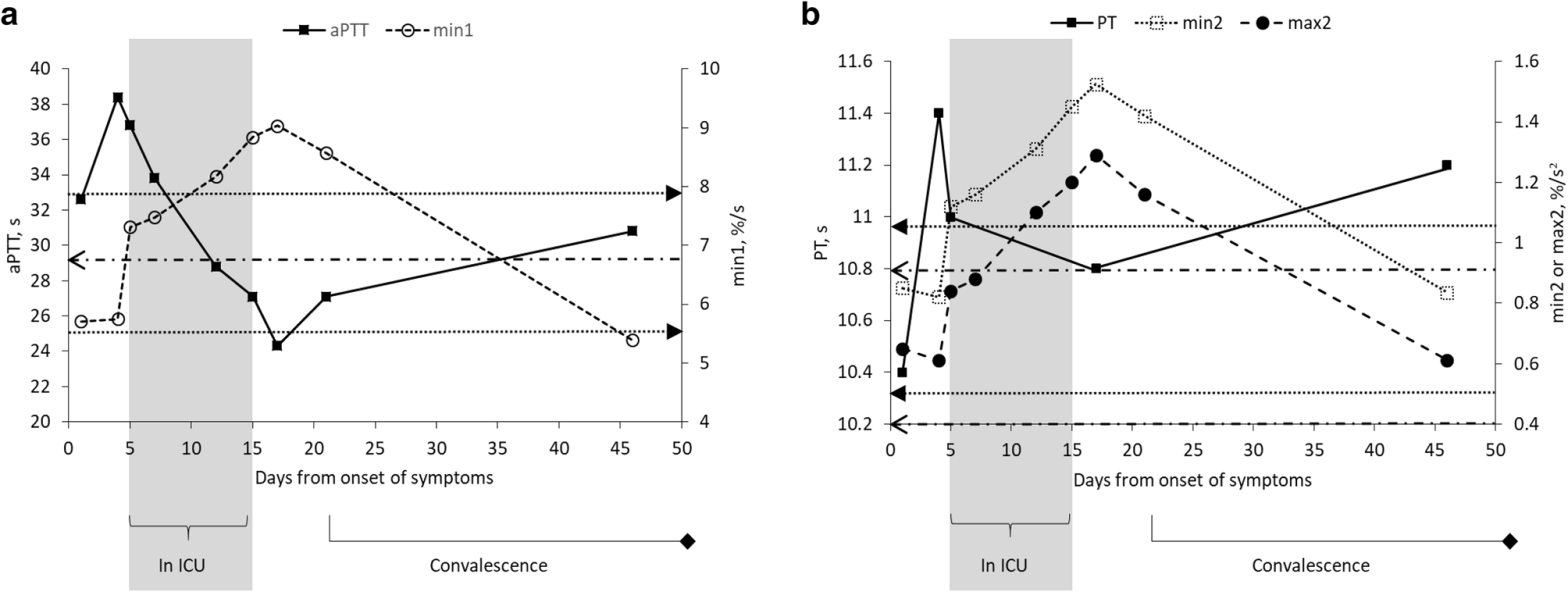
Dynamic changes of the serial haemostatic profiles of a COVID-19 patient from initial presentation to clinical deterioration* to subsequent recovery and convalescent phase. (**a**) Serial aPTT and min1 data of the patient. The horizontal dotted arrows ( ) depict the upper and lower limits of the reference intervals of aPTT while the horizontal dashed arrow ( ) shows the upper limit of min1 reference interval (min1 lower limit falls below the boundary of the graph shown). (**b**) Serial PT, min2 and max2 data of the patient. All the PT results fall within the reference intervals of 9.9 s to 11.4 s. The horizontal dashed arrows ( ) depict the upper and lower limits of the reference intervals of min2 while the horizontal dotted arrows ( ) show the upper and lower limits of max2 reference intervals. *Clinical deterioration defined as requiring intensive care unit (ICU) support

(b) CWA parameters increased with clinical deterioration of COVID-19 (Table 3 and Fig. 1)

**Table 3:** Inter-individual comparison of coagulation data (PT, aPTT and aPTT-based clot waveform analysis profiles) between patients with mild COVID-19^ and severe COVID-19^#^.

Coagulation Tests	Mild COVID-19 (n = 28)		Severe COVID-19 (n = 14)		*P* value
	Median (IQR)	Range	Median (IQR)	Range	
APTT, s	31.78 (3.04)	28.70–40.20	36.85 (7.38)	30.80–42.10	< 0.001
Min1, %/s	5.05 (1.38)	3.63–8.54	6.48 (1.23)	5.31–8.63	< 0.001
Min2, %/s^2^	0.74 (0.18)	0.53–1.32	0.92 (0.29)	0.67–1.31	0.033
Max2, %/s^2^	0.57 (0.13)	0.39–1.04	0.67 (0.22)	0.47–1.01	0.085
PT, s	10.33 (0.70)	9.70–13.20	10.85 (1.28)	9.50–13.00	0.180
D-dimer*, mg/L FEU	0.43 (0.64)	0.19–5.15	0.845 (0.61)	0.62–1.33	0.104
Fibrinogen^, g/L	3.08 (1.01)	2.23–3.44	4.56 (–)	4.12–6.81	0.057
Days since symptoms onset	7 (5)	4–34	9 (8)	4–24	0.218

* D-dimer results were only available in 11 and 4 patients with mild and severe COVID-19, respectively.

** Fibrinogen results were only available in 4 and 3 patients with mild and severe COVID-19, respectively.

^ Mild COVID-19 defined as patients who did not require supplementary oxygen support throughout the course of infection.

^#^ Severe COVID-19 defined as patients who required supplementary oxygen support during course of infection. All data were taken prior to the administration of prophylactic anticoagulant.

Comparing the PT, aPTT and CWA data between COVID-19 patients with clinically mild disease and COVID-19 patients who required supplementary oxygen support, a surrogate of more severe disease, aPTT was significantly more prolonged and CWA parameters, especially min1, were significantly elevated in patients with severe disease. PT and D-dimer results did not differ significantly between the two groups.

Serial PT, aPTT and CWA data depicting the various stages, from initial presentation through clinical deterioration to recovery and convalescence were available in one patient and the dynamic changes are shown in Fig. 1. Initial clinical deterioration was associated with prolongation of aPTT but aPTT normalised during the subsequent course of ICU stay and remained so at convalescent phase of COVID-19. However, min1 showed marked elevation upon ICU admission and continued to rise throughout the ICU stay despite normalisation of aPTT. Min1 only started to decrease at convalescence and eventual normalised on the 46th day from symptoms onset. Min2 and max2 demonstrated similar pattern of changes to min1 although the timing of their increment to the levels beyond the respective normal ranges was delayed compared to min1. This patient’s PT levels remained normal throughout.

(c) Critically ill COVID-19 patients CWA showed a trend of having higher CWA parameters as compared to critically ill patients with non-CoV-2-RV. (Table 4)

**Table 4:** Inter-individual comparison of coagulation data (PT, aPTT and aPTT-based clot waveform analysis profiles) between mild COVID-19 and non-CoV-2 respiratory viral infections^, as well as between critically ill COVID-19 and non-CoV-2 respiratory viral infections^#^.

Coagulation tests	Mild COVID-19 (n = 28)		Mild non-CoV-2 respiratory viruses (n = 3)		*P* value
	Median (IQR)	Range	Median (IQR)	Range	
APTT, s	31.78 (3.04)	28.70–40.20	29.60 (–)	25.80–33.30	0.229
Min1, %/s	5.05 (1.38)	3.63–8.54	5.76 (–)	3.60–7.79	0.729
Min2, %/s^2^	0.74 (0.18)	0.53–1.32	0.95 (–)	0.58–1.17	0.503
Max2, %/s^2^	0.57 (0.13)	0.39–1.04	0.80 (–)	0.47–0.95	0.385
PT, s	10.33 (0.70)	9.70–13.20	10.20 (–)	9.80–10.60	0.503
Days since symptoms onset	7 (5)	4–34	7 (–)	3–18	0.925

	Critically Ill COVID-19 (n = 9)		Critically Ill non-CoV-2 respiratory viruses (n = 2)		
APTT, s	40.20 (6.40)	33.30–42.10	41.45 (–)	29.70–53.20	1.000
Min1, %/s	6.27 (1.50)	5.31–8.63	4.57 (–)	3.17–5.97	0.327
Min2, %/s^2^	0.84 (0.30)	0.67–1.31	0.48 (–)	0.23–0.73	0.073
Max2, %/s^2^	0.60 (0.25)	0.47–1.01	0.39 (–)	0.21–0.57	0.218
PT, s	10.90 (1.20)	9.50–13.0	11.10 (–)	10.70–11.50	0.727
Platelet, × 10^9^/L	199 (127)	74–360	271 (–)	137–404	0.727
Days since symptoms onset	10 (11)	4–24	13 (–)	12–14	0.582

* No D-dimer and fibrinogen data presented as these results were not available in the mild non-CoV-2 respiratory viruses group.

^ Mild COVID-19 and non-CoV-2 respiratory viral infections defined as patients who did not require supplementary oxygen support throughout course of infection.

^#^ Critically ill COVID-19 and non-CoV-2 respiratory viral infections defined as patients who required intensive care unit support. All data were taken prior to the administration of prophylactic anticoagulant.

Patients with mild COVID-19 and those with non-CoV-2-RV had similar PT, aPTT and CWA profiles. In contrast, for patients who required ICU admission, critically ill COVID-19 patients CWA showed a trend of having higher CWA parameters compared to similar patients with non-CoV-2-RV, although their PT, aPTT and platelet counts were not different.

Paired analysis data, available in a limited number of subjects, comparing intra-individual CWA fluctuations before and after clinical deterioration demonstrated distinct pattern of change between COVID-19 and non-CoV-2-RV infections. In two non-CoV-2-RV patients, their median PT (10.65 s vs 11.10 s) and aPTT (37.30 s vs 41.45 s) became longer whereas their CWA profiles decreased (min1: 5.80%/s vs 4.57%/s; min2: 0.79%/s^2^ vs 0.48%/s^2^; max2: 0.58%/s^2^ vs 0.39%/s^2^) with clinical deterioration. The four pairs of data in COVID-19 patients also showed that their PT (10.35 s vs 11.20 s) and aPTT (40.20 s vs 41.40 s) were prolonged with clinical deterioration. However, in contrast to non-CoV-2-RV patients, their CWA parameters became elevated instead (min1: 5.82%/s vs 6.16%/s; min2: 0.74%/s^2^ vs 0.81%/s^2^; max2: 0.54%/s^2^ vs 0.62%/s^2^) with increasing severity of infection.

(d) CWA parameters correlated with D-dimer and fibrinogen

Based on ISTH recommendations, D-dimer of six folds the upper limit of normal in COVID 19 patients was a predictor of thrombotic events and thus may be used as a surrogate marker for consideration of higher dose of thromboprophylaxis^9^. Eight COVID-19 patients had D-dimer levels of greater than a cut-off of 3.00 mg/L FEU (approximately six folds of the upper normal limit). Of these, six patients had severe disease requiring oxygen support, one had mild disease and one was in convalescent phase of COVID-19. Of the two COVID-19 cases with thrombotic complications, only one had a high D-dimer of 4.33 mg/L FEU while another had a level of 1.22 mg/L FEU at the time when the thrombotic events occurred.

Amongst COVID-19 patients, 35 sets of data were available for correlation evaluation with D-dimer (Supplementary Table 2). While PT and aPTT did not correlate with D-dimer (R_S_ = 0.153, *P* = 0.143 and R_S_ = 0.183, *P* = 0.292, respectively), CWA parameters correlated significantly with D-dimer (min1: R_S_ = 0.476, *P* = 0.004; min2: R_S_ = 0.355, *P* = 0.037; max2: R_S_ = 0.282, *P* = 0.101). 21 sets of data were analysed for correlation with fibrinogen (Supplementary Table 2). With regards to fibrinogen, PT and aPTT showed no correlations (PT: R_S_ = -0.045, *P* = 0.847; aPTT: R_S_ = 0.269, *P* = 0.239) but all CWA parameters demonstrated significant positive correlations (min1: R_S_ = 0.812, *P* =  < 0.001; min2: R_S_ = 0.767, *P* < 0.001; max2: R_S_ = 0.695, *P* =  < 0.001).

## Discussion

The latest CHEST guidelines on VTE and its management in COVID -19 patients have provided more mature data on VTE rates^10^. Overall, venous thrombotic events are estimated to be around 8% in all COVID-19 patients and as high as 69% in critically ill patients. Other respiratory viral infections such as influenza are also known to be associated with thrombotic events^7,19^ with a 5.9% prevalence reported during the 2009 influenza pandemic^20^. Presently, indirect evidence and observations seem to suggest a higher thrombotic rate amongst COVID-19 patients compared to patients infected with other respiratory viruses^21^. However, direct comparison of thrombotic events between concurrent cohorts of COVID-19 and non-CoV-2-RV patients is lacking.

In contrast to other recent reports^2,22,23^, our findings did not show increased VTE events in either groups. This could be accounted by a number of factors peculiar to our study population. At the time of study, there was a large outbreak involving migrant workers residing in dormitories and all COVID-19 cases regardless of the severity were required to be hospitalized according to national policy and hence the majority of our patients were younger with less comorbidities and had a lower rate of ICU stay. Older age, presence of cardiovascular risk factors and ICU stays are all independent risk factors for VTE. In ICU patients with COVID-19, other studies have reported up to 69% VTE incidence^24,25^. The stark difference in our population may also suggests the influence of other factors such as racial predilection. Whilst local data show that the incidences of VTE are comparable amongst the major ethnic groups of Chinese, Indian and Malay^26^, Asians in general are purported to have lower risk of VTE compared to Caucasians^27–29^ although the actual reasons for this difference are still unclear. In our center, VTE screening was not done as a routine for asymptomatic cases. Confirmatory imaging for VTE would only be performed for symptomatic or clinically suspicious cases. As such our VTE detection rates may be lower compared to centers that practice routine screening of their patients. Albeit the variations in threshold or protocols for detecting VTE in the various studies, it is compelling that our thrombotic rates were clearly much lower compared to other centers.

COVID-19 and other acute respiratory viruses have both been reported to be associated with higher risk of arterial thrombotic events as well. Acute coronary syndrome is reported in no greater than 3% of acute influenza or other viral infections^5,30^ whilst in COVID-19, the rates vary from 3.4% to 11.2%^21,31^. We, however, reported low arterial event rates in our patient cohorts and the potential reasons accounting for these differences are likely to be similar to those discussed above for VTE. In our cohort, the arterial events occurred in patients who were critically ill. As none of the patients underwent a coronary angiogram, we were unable to conclusively establish the presence of atherothrombosis. Given that they were critically ill, myocardial infarction could have also been due to an acute mismatch between oxygen supply and demand and not a true thrombotic event. No ischemic events were reported in patients who had mild disease. Nonetheless, early recognition of cardiac complications in both COVID-19 and other viral infections cannot be over-emphasized to reduce morbidity and mortality.

We also evaluated the coagulation profiles of our COVID-19 patients across the different spectrum of disease severity. Commensurate with the absence of thrombotic events observed, none of our mild COVID-19 cases demonstrated evidence of DIC or SIC and their CWA parameters were not elevated. Marked elevation of D-dimer, defined as six-times upper limit of normal, was suggested by ISTH as a possible consideration for intensification of prophylactic pharmacological anticoagulation in COVID-19^9^. However, this cut-off was neither sensitive nor specific for thrombotic events in our cohort as none of our patients with markedly raised D-dimers developed venous thromboembolic event and only one of them suffered an arterial event. We also observed that aPTT but not PT was significantly different between patients with mild and severe COVID-19. In COVID-19, prolonged aPTT has been reported in more severe disease^8^ and in association with the presence of lupus anticoagulant, a prothrombotic marker^32,33^. However, no clotting factors and lupus anticoagulant assays were performed in our patients.

To further evaluate the thrombotic potential in COVID-19, we analysed available CWA parameters as a global haemostatic assessment. It has been reported that CWA parameters differ in various types of infections^34^ and increased CWA is associated with hypercoagulability^35,36^. Our findings suggest that there were no significant changes in the overall haemostatic functions in mild COVID-19 but severe COVID-19 was associated with a prothrombotic state and this hypercoagulability gradually normalizes during the convalescent phase. Although CWA parameters correlated with D-dimer and fibrinogen, the strengths of association were weak and moderate respectively and this suggests that the haemostatic functions measured by these assays do not replicate each other. Hence, composite data from these multiple assays might provide more insight into the overall haemostatic dysfunctions of COVID-19 patients. These assays may therefore complement each other to better risk stratify the thrombotic risk of COVID-19 patients which may guide thromboprophylaxis management. Nonetheless, the coagulation assays in this study only evaluated the plasmatic components of haemostasis. Endothelial functions and cellular components, including platelets and leucocytes, and fibrinolytic functions are not assessed but may contribute significantly to coagulation changes in COVID-19^33,37^.

ISTH has recommended for a universal strategy of routine thromboprophylaxis for all hospitalized COVID-19 patients with standard dose LMWH or unfractionated heparin after assessment of bleeding risk. While existing literature do suggest a significantly high rate of thrombotic events in COVID-19 patients, and understandably warrants the recommendation for thromboprophylaxis, our cohort study has not proven the case in our population. As routine thromboprophylaxis is not without risks, the adoption of this strategy for all hospitalised COVID-19 patients may not be universally suitable. Adjustments to these recommendations should be made taking into account the local population profile and prevalence of thrombotic events. Irrefutably, analysis of hemostatic parameters showed a hypercoagulable state in patients with more severe COVID-19 and thus supporting the use of thromboprophylaxis in critically ill COVID-19 patients.

Although our study has several limitations due to its retrospective nature and the number of thrombotic events in both groups were small thus limiting statistical comparison, we believe our comparison data are sufficiently robust as we included all consecutive patients with the same admitting criteria within the same study period and the demographics between both groups were comparable.

## Conclusion

The thrombotic rates in hospitalised COVID-19 and non-CoV-2-RV patients who were young with relatively few comorbidities and predominantly mild disease were low and did not differ significantly between the two groups. Hemostatic assays, in our study the use of CWA, did however demonstrate a trend of increased hypercoagulability in severely ill COVID-19 patients compared to severely ill non-CoV-2-RV patients. A more individualized thrombotic risk assessment and management approach, possibly involving the combination of various clinical factors and coagulation markers, is an area of unmet needs and warrants further research works.

## Supplementary Information

Supplementary Information.

## Data Availability

The data used and analyzed in this study are available from the corresponding author on reasonable request.
