# Supplementary material for: Clinical and laboratory features of hypercoagulability in COVID-19 and other respiratory viral infections amongst predominantly younger adults with few comorbidities
Source: Sci Rep. 2021 Jan 19;11:1793. doi: 10.1038/s41598-021-81166-y (PMC7815883; doi:10.1038/s41598-021-81166-y)
Supplement: Supplementary file 1 — Supplementary Information. [file 41598_2021_81166_MOESM1_ESM.docx]

**Title:** Clinical and laboratory features of hypercoagulability in COVID-19 and other respiratory viral infections amongst predominantly younger adults with few comorbidities.

**Running title: COVID 19 and thrombosis**

**Authors**

Chuen Wen Tan, MB BCh BAO, FRCPath^1^*; Jing Yuan Tan, MBBS^2^*; Wan Hui Wong, PhD^1^; May Anne Cheong, MRCP^1^; Ian Matthias Ng, MBBS^3^; Edwin Philip Conceicao, BSc^3^, Jenny Guek Hong Low, MBBS, MPH^4,5^; Heng Joo Ng, MBBS, FRCPath^1^; Lai Heng Lee, MBBS^1^.

*Contributed equally

Supplementary Table 1: Inter-individual comparison of coagulation data (PT, aPTT and aPTT-based clot waveform analysis profiles) between patients with mild COVID-19* and patients in convalescent phase.

| **Coagulation Tests** | **Mild COVID-19 (n=28)** | | **Convalescence (n=12)** | | **P-value** |
| --- | --- | --- | --- | --- | --- |
|  | **Median (IQR)** | **Range** | **Median (IQR)** | **Range** |  |
| APTT, s | 31.78 (3.04) | 28.70 – 40.20 | 30.68 (4.26) | 27.40 – 33.65 | 0.036 |
| Min1, %/s | 5.05 (1.38) | 3.63 – 8.54 | 5.39 (2.80) | 2.32 – 7.53 | 0.805 |
| Min2, %/s^2^ | 0.74 (0.18) | 0.53 – 1.32 | 0.83 (0.48) | 0.38 – 1.22 | 0.389 |
| Max2, %/s^2^ | 0.57 (0.13) | 0.39 – 1.04 | 0.66 (0.38) | 0.31 – 1.04 | 0.313 |
| PT, s | 10.33 (0.70) | 9.70 – 13.20 | 10.40 (1.16) | 9.70 – 11.60 | 0.475 |
| Days since symptoms onset | 7 (5) | 4 - 34 | 53 (36) | 16 – 100 | <0.001 |

* Mild COVID-19 defined as patients who did not require supplementary oxygen support throughout the course of infection.

Supplementary Table 2: Coagulation data (PT, aPTT, aPTT-based clot waveform analysis profiles) with D-dimer and fibrinogen, respectively, amongst COVID-19 patients available for correlation evaluation.

| **Coagulation tests (n=35*)** | **Median (IQR)** | **Range** | **Correlation with D-dimer** | | **Coagulation tests (n=21^)** | **Median (IQR)** | **Range** | **Correlation with fibrinogen** | |
| --- | --- | --- | --- | --- | --- | --- | --- | --- | --- |
|  |  |  | **R_s_** | **P-value** |  |  |  | **R_s_** | **P-value** |
| D-dimer, mg/L FEU | 0.88 (2.19) | 0.19 – 31.47 |  |  | Fibrinogen, g/L | 3.28 (1.80) | 1.41 – 6.81 |  |  |
| aPTT, s | 32.15 (9.90) | 26.60 – 54.70 | 0.183 | 0.292 | aPTT, s | 29.50 (4.42) | 25.80 – 42.10 | 0.269 | 0.239 |
| Min1, %/s | 5.76 (2.08) | 2.32 – 8.63 | 0.476 | 0.004 | Min1, %/s | 5.37 (2.55) | 2.32 – 8.63 | -0.045 | 0.847 |
| Min2, %/s^2^ | 0.79 (0.33) | 0.38 – 1.31 | 0.355 | 0.037 | Min2, %/s^2^ | 0.77 (0.40) | 0.38 – 1.31 | 0.812 | <0.001 |
| Max2, %/s^2^ | 0.60 (0.26) | 0.31 – 1.01 | 0.282 | 0.101 | Max2, %/s^2^ | 0.61 (0.32) | 0.31 – 1.01 | 0.767 | <0.001 |
| PT, s | 10.80 (1.00) | 9.80 – 15.20 | 0.153 | 0.143 | PT, s | 10.90 (1.15) | 10.00 – 13.60 | 0.695 | <0.001 |

*35 D-dimer samples from 24 patients. ^21 fibrinogen samples from 17 patients.
